# Reduced Height (*Rht*) Alleles Affect Wheat Grain Quality

**DOI:** 10.1371/journal.pone.0156056

**Published:** 2016-05-19

**Authors:** Richard Casebow, Caroline Hadley, Rajneet Uppal, Molla Addisu, Stefano Loddo, Ania Kowalski, Simon Griffiths, Mike Gooding

**Affiliations:** 1 School of Agriculture, Policy and Development, University of Reading, Reading, Berkshire, United Kingdom; 2 Department of Environmental Agronomy and Crop Sciences, University of Padova, Legnaro, Padova, Italy; 3 John Innes Centre, Norwich, Norfolk, United Kingdom; 4 Institute of Biological, Environmental and Rural Sciences, University of Aberystwyth, Aberystwyth, Ceredigion, United Kingdom; Institute of Genetics and Developmental Biology, CHINA

## Abstract

The effects of dwarfing alleles (reduced height, *Rht*) in near isogenic lines on wheat grain quality are characterised in field experiments and related to effects on crop height, grain yield and GA-sensitivity. Alleles included those that conferred GA-insensitivity (*Rht-B1b*, *Rht-B1c*, *Rht-D1b*, *Rht-D1c*) as well as those that retained GA-sensitivity (*rht*(tall), *Rht8*, *Rht8* + *Ppd-D1a*, *Rht12*). Full characterisation was facilitated by including factors with which the effects of *Rht* alleles are known to interact for grain yield (i.e. system, [conventional or organic]; tillage intensity [plough-based, minimum or zero]; nitrogen fertilizer level [0–450 kg N/ha]; and genetic backgrounds varying in height [cvs Maris Huntsman, Maris Widgeon, and Mercia]. Allele effects on mean grain weight and grain specific weight were positively associated with final crop height: dwarfing reduced these quality criteria irrespective of crop management or GA-sensitivity. In all but two experiments the effects of dwarfing alleles on grain nitrogen and sulphur concentrations were closely and negatively related to effects on grain yield, e.g. a quadratic relationship between grain yield and crop height manipulated by the GA-insensitive alleles was mirrored by quadratic relationships for nitrogen and sulphur concentrations: the highest yields and most dilute concentrations occurred around 80cm. In one of the two exceptional experiments the GA-insensitive *Rht-B1b* and *Rht-B1c* significantly (*P*<0.05) reduced grain nitrogen concentration in the absence of an effect on yield, and in the remaining experiment the GA-sensitive *Rht8* significantly reduced both grain yield and grain nitrogen concentration simultaneously. When *Rht* alleles diluted grain nitrogen concentration, N:S ratios and SDS-sedimentation volumes were often improved. Hagberg falling number (HFN) was negatively related to crop height but benefits from dwarfing were only seen for GA-insensitive alleles. For HFN, therefore, there was the strongest evidence for a direct pleiotropic effect of GA-insensitivity, rather than an effect consequential to yield and/or height.

## Introduction

Improvements in wheat grain yield since in the 1960s are frequently ascribed to ‘semi-dwarfing’, i.e. reducing post-anthesis crop height to less than 1.0m. This reduction improves harvest index; reduces lodging risk in fertile and humid conditions; and so increases responses to nitrogen availability associated with improved light interception [1]. By the 1990’s major reduced height (*Rht*) alleles were present in about 80% of registered wheat cultivars; in 90% of those cases either *Rht-B1b* or *Rht-D1b* was responsible for semi-dwarfing [2]. These *Rht-X1x* alleles contain mutations in DELLA proteins [3–4] which repress gibberellic acid (GA)-responsive growth [4–5]. More severe dwarfing is possible with alternative alleles at both loci, represented here by *Rht-B1c* and *Rht-D1c* [4, 6, 7].

Although effects of GA-insensitivity on grain yield are well known [6–7] the responses of grain quality to reduced height are less well characterised. Direct pleiotropic effects of some *Rht-X1x* alleles have been suggested for grain protein concentration dilution [8] and alpha-amylase activity [9], and GA activity has been linked to effects on grain size [10]. The purpose here is to fully characterise the effects of GA-insensitivity alleles on common measures of wheat grain quality by assessing criteria in experiments that include treatment factors known to influence the effect of *Rht-X1x* alleles on grain yield (final crop height [6,7], cropping system [7], tillage intensity [11, 12] and nitrogen fertilizer level [13]). Assessing the evidence for pleiotropic effects of GA-insensitivity is facilitated by the inclusion GA-sensitive alleles i.e. the semi-dwarfing *Rht8* which possibly interferes with brassinosteroid-mediated responses [14] and is commonly linked to the photoperiod insensitivity allele *Ppd-D1a* in South European wheats when early meiosis and flowering is needed to escape excessive summer heat and drought [15]; and the severe dwarfing *Rht12* [16] that has been tentatively proposed to confer impeded GA biosynthesis [17].

Grain quality characters assessed included: mean grain weight and specific (or test-) weight as crude indicators of milling performance [18–19]; Hagberg falling number as an assessment of *alpha*-amylase activity [20–21] which if present at excessive levels can impede loaf manufacture and quality [19]; concentrations of nitrogen and sulphur because of their influence on loaf quality [19, 22–23]; and SDS-sedimentation volume tests [24] as a small-scale assessment of baking potential that is reliant on glutenin subunits [25–26] and disulphide bonds [27].

## Material and Methods

### Near Isogenic Lines

The near isogenic lines varying for dwarfing allele and background used in this study are listed in Table 1. Lines were developed by repeated back crossing in to the background cultivar after the progeny had been variously marked and selected depending on the allele [2, 6, 28, 29, 30]. With respect to the backgrounds, Mercia was introduced commercially in 1983, and was the last widely-used non-semi-dwarf winter wheat cultivar suited for bread making in the UK. However, the stature of Mercia was comparable to many of its contemporary cultivars containing *Rht1-B1b* or *Rht1-D1b*. NILs in older and taller backgrounds were, therefore, also included: Maris Widgeon (introduced in 1964 with good bread making potential) and Maris Huntsman (introduced in 1969 with poor breadmaking potential). In experiment 5 (Table 1) the background was Paragon: released in 1999, Paragon is a spring wheat with good breadmaking potential but with a comparatively tall stature when sown in the autumn as was the case here. With respect to the GA-insensitive alleles: *Rht1-B1b* and *Rht1-D1b* were from ‘Norin 10’; *Rht-B1c* was from ‘Tom thumb’; and *Rht-D1c* was from ‘Ai-Bian’. The GA-sensitive alleles comprised *Rht8* + *Ppd-D1a* from ‘Mara’ and *Rht12* from ‘Karcagi 522’.

**Table 1:** Treatments in different experiments and years.

Harvest year	Blocks	Background	*Rht* alleles in near isogenic lines	Levels of other factors	Yield refe-rence
**Series 1: Conventional**					
2006	4	Mercia	*1a*(tall), *B1b*, *B1c*, *D1b*, *D1c*, *8+Ppd-D1a*, *12*		7
2007	4	Mercia	*1a*(tall), *B1b*, *B1c*, *D1b*, *D1c*, *8+Ppd-D1a*, *12*		7
2008	3	Mercia	*1a*(tall), *B1b*, *B1c*, *D1b*, *D1c*, *8+Ppd-D1a*, *12*		7
		M. Huntsman	*1a*(tall), *B1b*, *B1c*, *D1b*, *B1b+D1b*, *B1c+D1b*		
		M. Widgeon	*1a*(tall), *B1b*, *B1c*, *D1b*, *B1b+D1b*, *B1c+D1b*		
2009	5	Mercia	*1a*(tall), *B1b*, *B1c*, *D1b*, *D1c*, *8+Ppd-D1a*, *12*		
		M. Huntsman	*1a*(tall), *B1b*, *B1c*, *D1b*, *B1b+D1b*, *B1c+D1b*		
		M. Widgeon	*1a*(tall), *B1b*, *B1c*, *D1b*, *B1b+D1b*, *B1c+D1b*		
2010	6	Mercia	*1a*(tall), *B1b*, *B1c*, *D1b*, *D1c*, *8+Ppd-D1a*, *12*		
		M. Huntsman	*1a*(tall), *B1b*, *B1c*, *D1b*, *B1b+D1b*, *B1c+D1b*		
		M. Widgeon	*1a*(tall), *B1b*, *B1c*, *D1b*, *B1b+D1b*, *B1c+D1b*		
2011	7	Mercia	*1a*(tall), *B1b*, *B1c*, *D1b*, *D1c*, *8+Ppd-D1a*, *12*		
		M. Huntsman	*1a*(tall), *B1b*, *B1c*, *D1b*, *B1b+D1b*, *B1c+D1b*		
		M. Widgeon	*1a*(tall), *B1b*, *B1c*, *D1b*, *B1b+D1b*, *B1c+D1b*		
**Series 2: Organic**					
2006	4	Mercia	*1a*(tall), *B1b*, *B1c*, *D1b*, *D1c*, *8+Ppd-D1a*, *12*		7
2007	4	Mercia	*1a*(tall), *B1b*, *B1c*, *D1b*, *D1c*, *8+Ppd-D1a*, *12*		7
2008	3	Mercia	*1a*(tall), *B1b*, *B1c*, *D1b*, *D1c*, *8+Ppd-D1a*, *12*		7
		M. Huntsman	*1a*(tall), *B1b*, *B1c*, *D1b*, *B1b+D1b*, *B1c+D1b*		
		M. Widgeon	*1a*(tall), *B1b*, *B1c*, *D1b*, *B1b+D1b*, *B1c+D1b*		
**Series 3: Tillage**					
2010	3	Mercia	*1a*(tall), *B1b*, *B1c*, *D1b*, *D1c*, *8+Ppd-D1a*, *12*	Plough-based, minimum, zero tillage	12
		M. Widgeon	*1a*(tall), *D1b*, *B1c*		
2011	3	Mercia	*1a*(tall), *B1b*, *B1c*, *D1b*, *D1c*, *8+Ppd-D1a*, *12*	Plough-based, minimum, zero tillage	12
		M. Widgeon	*1a*(tall), *D1b*, *B1c*		
**Series 4: N rate**					
2010	3	Merica	*B1a*(tall), *B1b*, *B1c*	0, 100, 200 and 350 kg N ha^-1^	13
		M. Huntsman	*B1a*(tall), *B1b*, *B1c*		
		M. Widgeon	*B1a*(tall), *B1b*, *B1c*		
2011	3	Merica	*B1a*(tall), *B1b*, *B1c*	0, 50, 100, 200, 350, 450 kg N ha^-1^	
		M. Widgeon	*B1a*(tall), *B1b*, *B1c*		
**Series 5: N rate x *Rht8***					
2014	5	Paragon	Paragon, *rht*(tall), *Rht8*	40, 100, 200 kg N ha^-1^	30

### Site details and crop husbandry

Five series of randomized block experiments (Table 1) were undertaken at the Crops Research Unit, Sonning, University of Reading, UK (51° 29’ N, 0° 56’ W). All experiments: followed a 2 to 3 year sward of grass (*Lolium perenne*, *Dactylis glomerata*) and clover (*Trifolium repens*, *T*. *pretense*); were sown between 21 September and 11 November; and harvested between 2 August and 3 September. Crop development was typical of UK winter wheat with externally-visible stem extension starting in March, flag leaf emergence in May, anthesis in June, end of grain filling in July, and harvest maturity in August. For the seven years of experiments the average rainfall for successive months from March to August inclusive was 41, 30, 55, 53, 55, 71 mm; mean temperature was 6.7, 10.0, 12.4, 15.6, 17.5, 16.3°C.

Except when forming part of the treatment structure, the land was prepared by inversion ploughing to 300 mm, followed by power harrowing (Lely Roterra). Untreated seeds (300 m^-2^, except 250 m^-2^ in 2008) were sown with a Hege 80 plot seed drill at 50 mm depth into 120 mm rows in 2 m wide plots (or sub-plots), separated by a 500 mm double track wheeling. Plot (or sub-plot) lengths were at least 6m. Herbicide applications were applied at growth stage (GS [31]) 19 and/or 31–32; and fungicide applications at GS 30–31, 39 and 59. No plant growth regulators were applied. In each year, 100 kg N ha^-1^ + 40 kg S ha^-1^ was applied as a mixture of granular ammonium nitrate and ammonium sulphate at GS 30–31. A further 100 kg N ha^-1^ was applied as ammonium nitrate between GS 34–39.

### Crop management treatment factors

For Series 2 (Table 1), a quarter of the experimental field site had been managed organically since 2001 [32] with no application of synthetic agrochemicals or fertilizers.

In Series 3, tillage main plots (50 m × 5 m) were randomized in blocks and divided into ten randomized sub-plots (2.5 m × 10 m) to receive the different NILs. In this series only the first sowing followed the grass plus clover ley; the second sowing superimposed the same treatments onto those of the first. In the minimum tillage plots there was no primary cultivation but a surface tilth (20–30 mm) was achieved with a single shallow pass with the power harrow and seeds drilled to a nominal depth of 30 mm. In the zero-tilled plots, seeds were roughly released into coulter slots formed by the drill.

In Series 4, main plots comprised the background × allele combinations; each containing randomized sub-plots allocated to receive different rates of N fertilizer applied as granular ammonium nitrate. In 2009/10 for the three sub-plots receiving N, 50 kg N ha^-1^ was applied at GS 31 and again at flag leaf emergence (GS 39). The 200 and 350 kg N ha^-1^ treatments received a further 100 and 250 kg N ha^-1^ respectively at the second node stage (GS 32). In 2010/11 the total N rates were applied in equal splits at GS 31 and 33.

In Series 5 the nitrogen application rates comprised the mainplots which were divided into NIL sub-plots. All plots received 40 kg N ha^-1^ at GS 30–31. For the 100 and 200 kg N ha^-1^ treatments the required additional fertilizer was applied at GS 34–39.

### Assessments

Crop height was calculated as the mean of three measurements with a rising disc of polystyrene [33]: at anthesis, the end of grain filling, and at harvest maturity. The central portion of each plot was combine-harvested at maturity with a 1.3 m cutter bar. Mean grain weights were determined from a divided sample of at least 250 grains per plot. Specific weight (SW) was measured using a chondrometer calibrated to ISO 7971:1995. Grain samples (20 g per plot) were dried at 80°C for 48 h to determine moisture content, and to adjust yields and mean grain weights to a dry matter basis. Samples of fresh grain (100 g per plot) were milled using a Laboratory Mill 3100 (Perten Instruments AB, Huddinge, Sweden) and tested for HFN with a Perten Instruments Falling Number 1500 machine assessed to ISO 3039. The nitrogen concentration was determined on dried flour with an oxidative combustion method using an automated Dumas type analyser (Leco FP-528; Leco Instruments (UK) Ltd., Stockport, Cheshire, UK). Grain sulphur content was also determined after oxidative combustion with a Leco SC-144DR. The SDS sedimentation test was performed as an indicator of potential baking performance (BSI ISO/CD 309).

### Statistical analysis

Statistical procedures were performed using GENSTAT (VSN International, Hemel Hempstead, UK). For Series 1 and 2 plot data were subjected to analysis of Residual Maximum Likelihood (REML) with a fixed model of NIL (i.e. each background x allele combination) and a random model of Year/Block/Plot. To investigate responses of grain quality variates to height effects mediated through GA-insensitivity, quadratic regression was fitted to data with alleles varying at the *Rht-X1x* loci. The fitted model was pol(height;2)+background. For presentation purposes the constants for the different backgrounds were removed to rebase the NIL grain quality means (including *Rht8* and *Rht12*, in addition to *Rht-X1x* alleles) to those of Maris Huntsman (Background x Allele means before rebasing are available in S1 and S2 Tables).

Series 3 data were subjected to analyses of variance comprising a treatment structure of NIL x Tillage and a block structure of Block / Mainplot / Plot / Year. Relationships with height were investigated as for Series 1 and 2, rebasing quality means to Mercia (Background x Allele means before rebasing are available in S3 Table).

In Series 4, within year analyses of variance (ANOVA) included a treatment structure of Background * Allele * pol(N rate;2) to split N effects into polynomial contrasts. Allele x N rate means are presented in the results; Background x Allele x N rate means for each year are presented in S4 and S5 Tables.

For Series 5 the ANOVA comprised a treatment structure of NIL * pol(N rate; 1) and a Block structure of block / N rate / NIL.

## Results

### Grain yield

**Fig 1:**
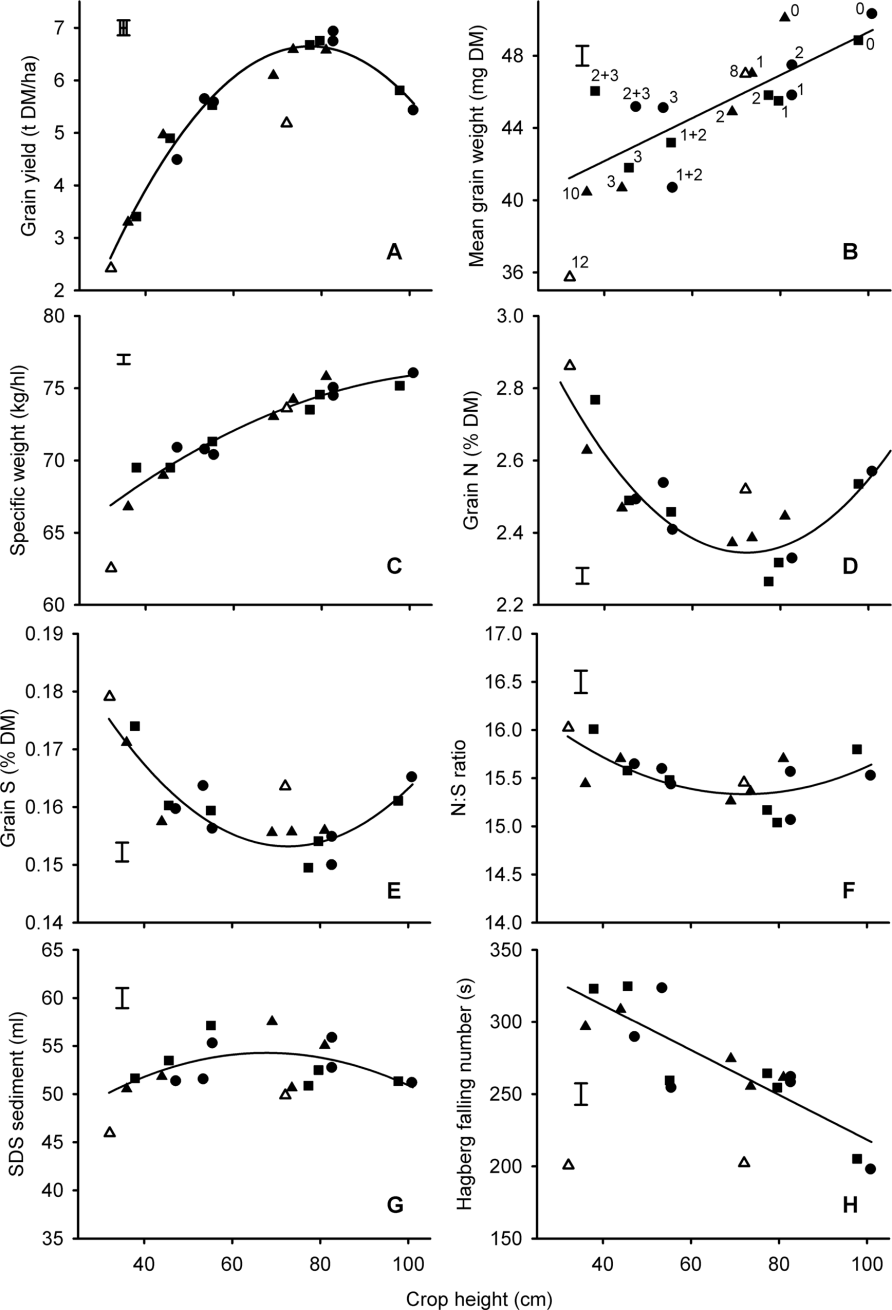
*Rht* effects on yield and quality of conventional wheat related to crop height. Numbers in panel B correspond to *Rht-* alleles (0 = tall, 1 = *B1b*, 2 = *D1b*, 3 = *B1c*, 8 = *Rht8* + *Ppd-D1a*, 10 = *D1c*, 12 = *Rht12*) in Mercia (▲, △), Maris Huntsman (■) and Maris Widgeon (●) backgrounds. Fits are quadratic or linear. Open symbols (8 = *Rht8* + *Ppd-D1a*, 12 = *Rht12*) are gibberellin-sensitive dwarfing alleles and not included in the fits. Alleles in all other panels can be inferred from labelling in panel B as heights of alleles are consistent. Main effects of background on the fitted constant have been removed from all data points. Points are means from 29 year x block combinations. Error bars are one standard error of difference between means (>300 D.F.).

We demonstrate here over the six experiments in Series 1 that the response of grain yield to height in the range of 30 cm–110 cm, as modified by *Rht-X1x* alleles can be very accurately described by a quadratic response (*r*^*2*^_*adj*_ = 0.95, Fig 1A). With this conventional management, the optimum height was around 80 cm whether achieved by adding *Rht-B1b* or *Rht-D1b* to the otherwise excessively tall backgrounds of Maris Huntsman or Maris Widgeon, or by retaining the *rht*(tall) allele in the shorter background of Mercia. Also, effects of different alleles and background combinations on yield are similar at similar heights e.g. Maris Huntsman *Rht-B1b* + *Rht-D1b*, and Maris Widgeon *Rht-B1b* + *Rht-D1b* and *Rht-B1c* had similar heights and produced near-identical effects on yield; Mercia *Rht-B1c*, Maris Huntsman *Rht-B1c* and Maris Widgeon *Rht-D1b* + *Rht-B1c* were similarly clustered.

**Fig 2:**
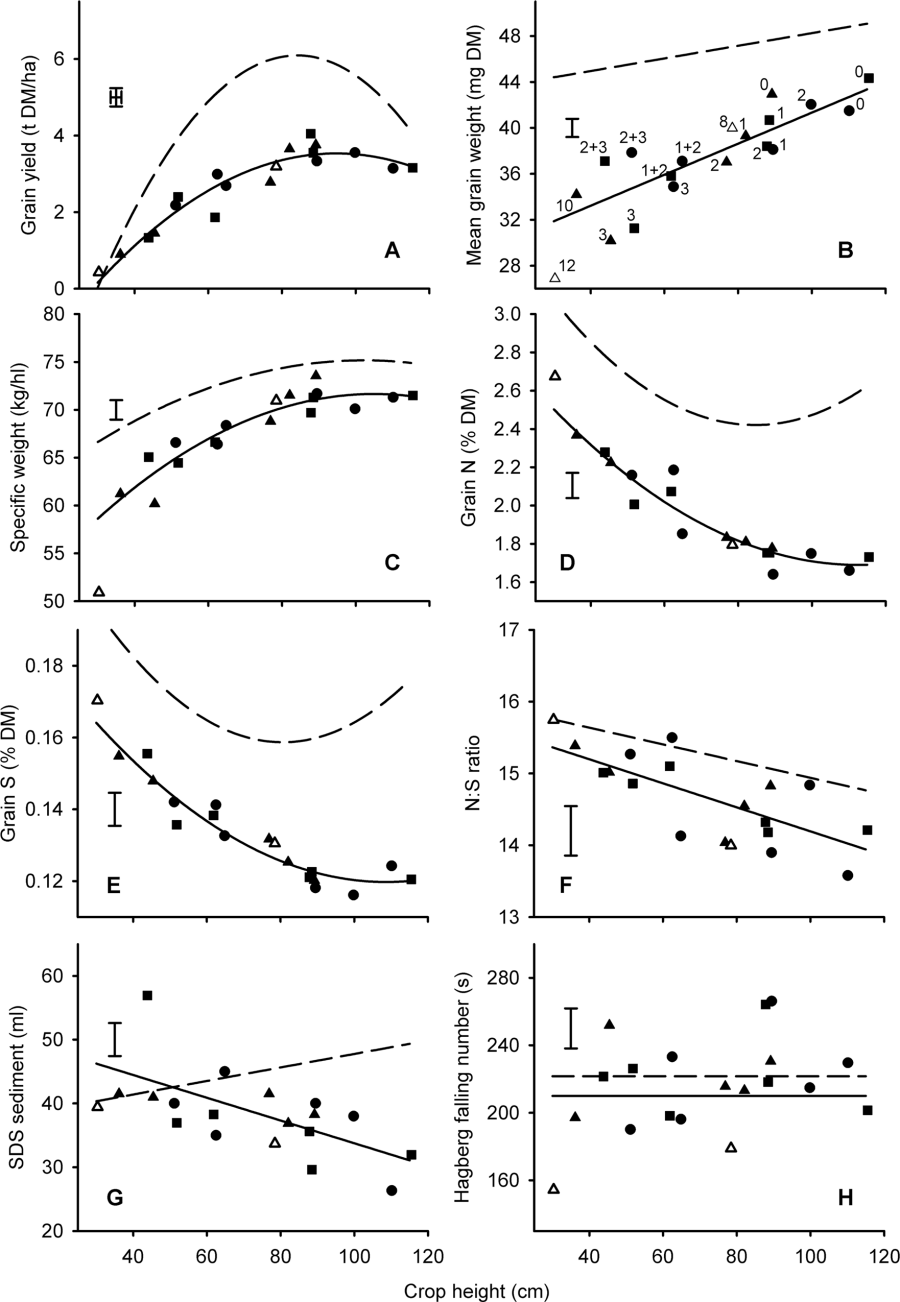
*Rht* effects on yield and quality of organic wheat related to crop height. Numbers in panel B correspond to *Rht-* alleles (0 = tall, 1 = *B1b*, 2 = *D1b*, 3 = *B1c*, 8 = *Rht8* + *Ppd-D1a*, 10 = *D1c*, 12 = *Rht12*) in Mercia (▲, △), Maris Huntsman (■) and Maris Widgeon (●) backgrounds. Fits are quadratic or linear. Open symbols (8 = *Rht8* + *Ppd-D1a*, 12 = *Rht12*) are gibberellin-sensitive dwarfing alleles and not included in the fits. Alleles in all other panels can be inferred from labelling in panel B as heights of alleles are consistent. Main effects of background on the fitted constant have been removed from all data points. Points are means from 11 year x block combinations. Error bars are one standard error of difference between means (>80 D.F.). Dotted lines are fits to the same lines managed conventionally at the same site in the same years.

**Fig 3:**
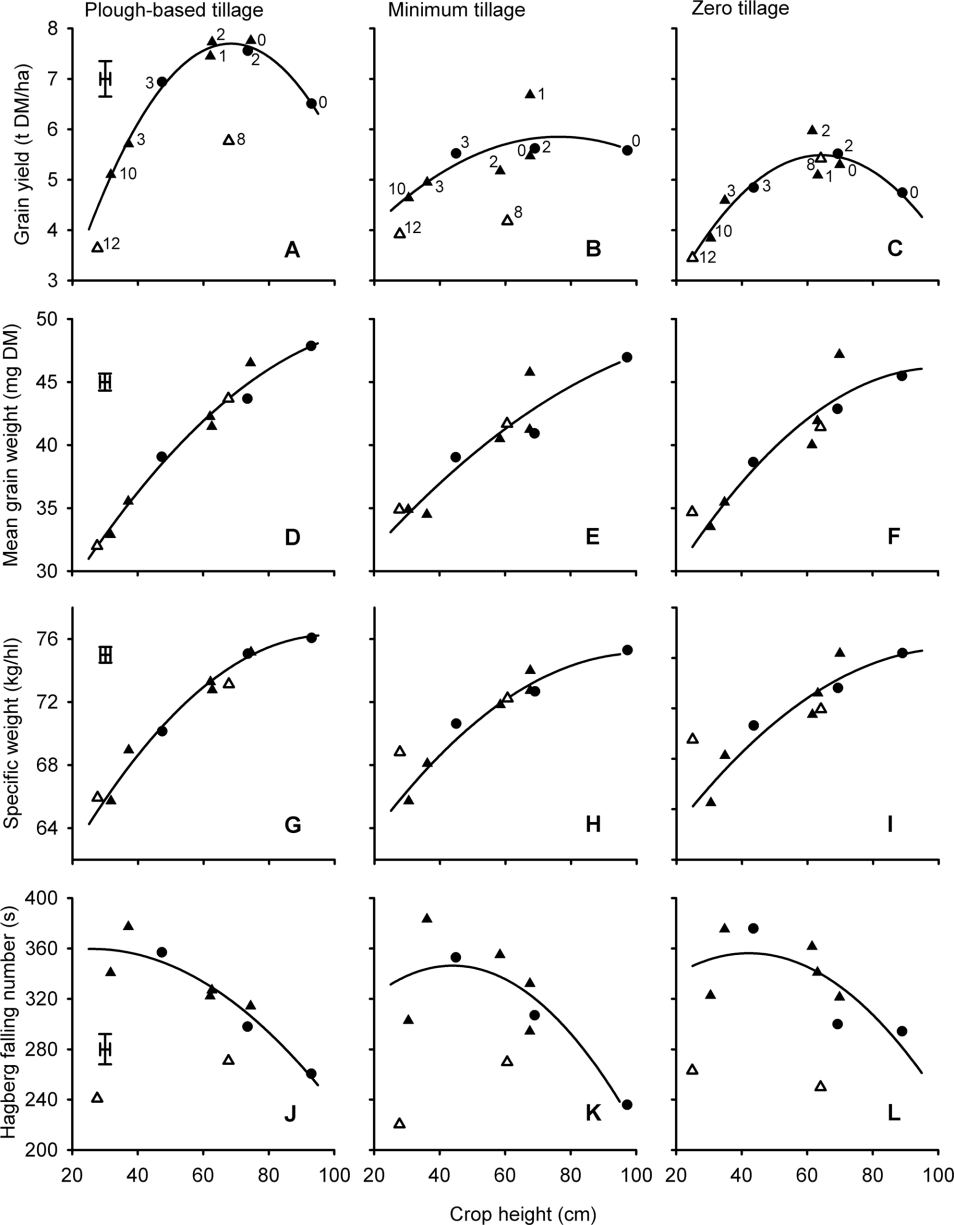
*Rht* and tillage effects on yield and quality of wheat related to crop height. Numbers in panels A-C correspond to *Rht-* alleles (0 = tall, 1 = *B1b*, 2 = *D1b*, 3 = *B1c*, 8 = *Rht8* + *Ppd-D1a*, 10 = *D1c*, 12 = *Rht12*) in Mercia (▲, △), and Maris Widgeon (●) backgrounds. Fits are quadratic. Open symbols (8 = *Rht8* + *Ppd-D1a*, 12 = *Rht12*) are gibberellin-sensitive dwarfing alleles and not included in the fits. Alleles in all other panels can be inferred from labelling in panels A-C as heights of alleles are consistent. Main effects of background on the fitted constant have been removed from all data points. Points are means from 3 blocks in each of two years. Error bars are one standard error of difference for comparing alleles within tillage treatment (54 D.F.).

There was no significant penalty for heights in excess of 80 cm in the organic system (Fig 2A) or in the minimum tillage context (Fig 3B) although in this latter case, the data were more variable.

**Fig 4:**
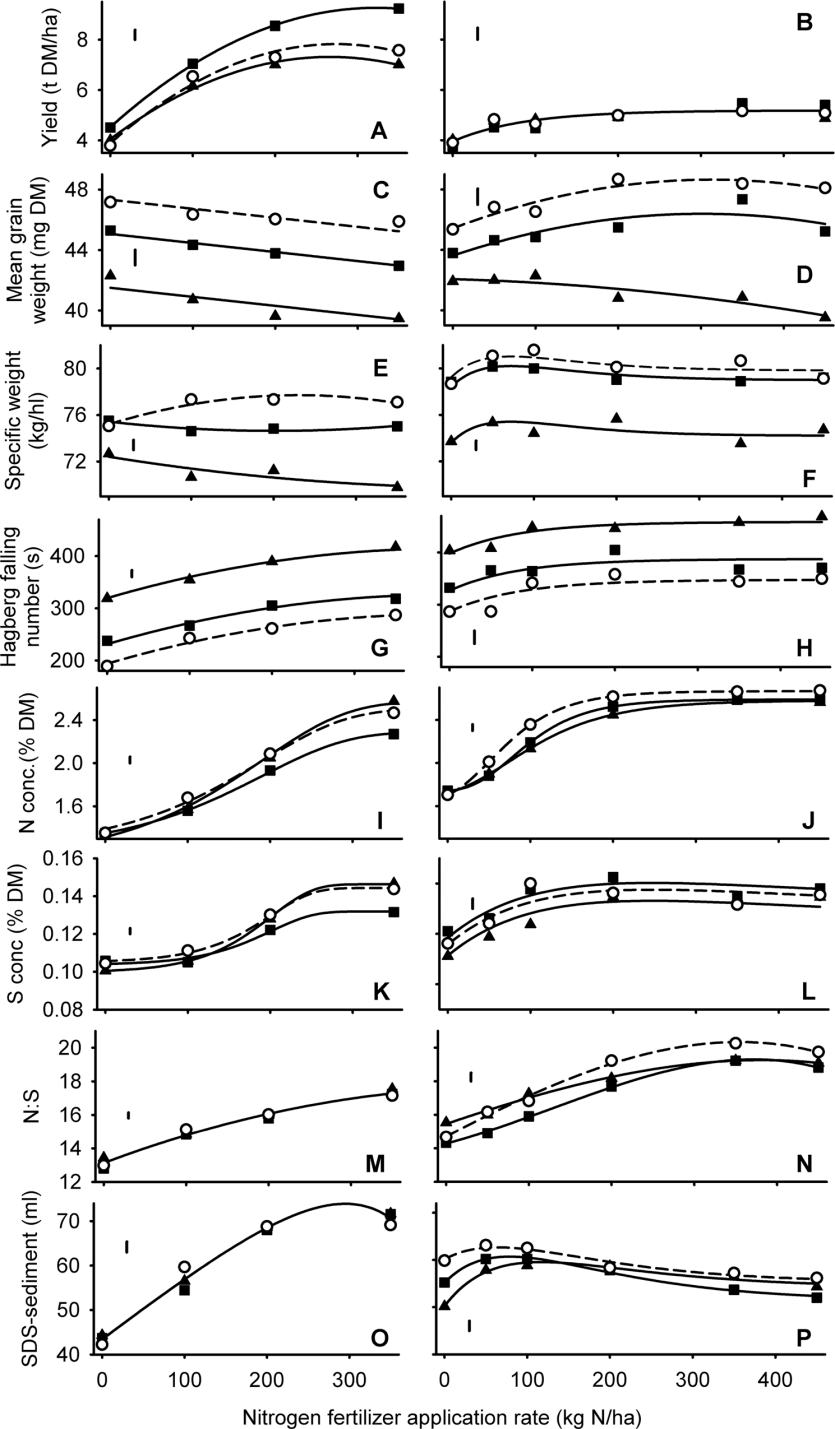
*Rht* allele and nitrogen effects on yield and quality of wheat. Allele at the *Rht-B1* locus denoted: ○, dashed line = tall; ▲ = *-B1b*; ● = *-B1c*. Error bars are one standard error of difference (D.F. > 50) for comparing alleles at the same N level. Left and right panels are for grain harvested in 2010 and 2011 respectively.

Grain yield was much more responsive to nitrogen fertilizer application in 2010 than in 2011 (Fig 4A and 4B). There was a highly significant (*P<*0.001) Allele x N rate interaction in 2010 because the grain yield of *Rht-B1b* was more responsive to N rate than either *rht*(tall) or *Rht-B1c* (Fig 4A). There was no main effect of Allele on grain yield in 2011 (*P* = 0.997).

*Rht8*+*Ppd-D1a* significantly reduced grain yield in the conventional experiments when compared with other lines of comparable height (Fig 1A). *Rht8*, when isolated from *Ppd-D1a* also reduced yields compared with taller lines in Paragon when grown at higher rates of nitrogen (Table 2). The negative effect of *Rht8*+*Ppd-D1a* disappeared in the organic experiments (Fig 2A), and also following zero tillage (Fig 3C). Similarly, *Rht8* was not detrimental to yield in a Paragon background in the 40Kg N ha^-1^ treatment (Table 2).

**Table 2:** Effect of *Rht8* and nitrogen fertilizer application on the grain quality of wheat.

40 kg N ha^-1^			100 kg N ha^-1^			200 kg N ha^-1^			Standard error of difference
Paragon	*rht*(tall)	*Rht8*	Paragon	*rht*(tall)	*Rht8*	Paragon	*rht*(tall)	*Rht8*	(24 D.F.)
Height (cm)									
100	104	94	107	111	102	110	114	106	1.1
Grain yield (t DM ha^-1^)									
5.70	5.47	5.72	7.25	7.39	6.97	9.16	9.22	8.75	0.157
Mean grain weight (mg DM)									
49.0	50.5	48.6	48.5	49.6	50.0	49.0	49.8	49.2	0.73
Specific weight (kg hl^-1^)									
79.6	79.8	78.4	79.4	79.5	77.7	79.7	80.4	78.0	0.64
Hagberg falling number (s)									
349	357	356	363	368	367	380	374	388	9.46
Nitrogen (% DM)									
1.52	1.52	1.53	1.57	1.54	1.55	1.91	1.88	1.83	0.019
Sulphur (% DM)									
0.104	0.106	0.106	0.106	0.107	0.103	0.118	0.117	0.115	0.0018
N:S ratio									
14.6	14.3	14.4	14.8	14.5	15.1	16.2	16.1	16.0	0.23
SDS-sedimentation (ml)									
56.2	51.6	55.8	61.4	56.2	58.4	78.4	73.6	73.8	1.94

*Rht12* produced the shortest plants. The grain yield of *Rht12* was broadly consistent with expectations from the fit to the *Rht-X1x* alleles irrespective of management or tillage (Figs 1A, 2A and 3A–3C)

### Mean grain weight

For the *Rht-X1x* alleles mean grain weight was positively related to height (Fig 1B) irrespective of production (Fig 2B) or tillage (Fig 3D–3F) system. The same effect was evident in the nitrogen response experiments (Fig 4C and 4D) where mean grain weight ranked *rht*(tall) > *Rht-B1b* > *Rht-B1c* for all nitrogen levels in both years.

With regards to the GA-sensitive alleles, *Rht8*+*Ppd-D1a* often reduced mean grain weight relative to *rht*(tall) (Figs 1B, 2B and 3D–3F) in accordance with the predicted effect of height from the *Rht-X1x* alleles. However, when isolated from *Ppd-D1a*, *Rht8* in Paragon did not reduce mean grain weight (Table 2). The severe dwarf, *Rht12*, often caused the biggest reduction in mean grain weight. This effect was sometimes (Figs 1B and 2B), but not always (Fig 3D–3F), more marked than the predicted effects of height from the *Rht-X1x* alleles.

### Specific weight

The effects of *Rht-X1x* allele on specific weight were consistently and accurately described by quadratic relationships: the penalty of incremental dwarfing becoming more exaggerated as height declined (Figs 1C, 2C and 3G–3I). The effect of *Rht-B1x* alleles interacted with nitrogen application rate (*P*<0.05; Fig 4E and 4F): on average, specific weight ranked *rht*(tall) > *Rht-B1b* > *Rht-B1c*, but *Rht-B1b* never led to a significantly lower specific weight than *rht*(tall) at the 0 N rate.

The effects of *Rht8* + *Ppd-D1a* were well described by effects of height as predicted by the fit for the *Rht-X1x* alleles (Figs 1C, 2C and 3G–3I). In the Paragon background, *Rht8* reduced grain specific weight (Table 2). In the conventional and organic experiments the negative effect of *Rht12* on grain specific weight was particularly marked (Figs 1C and 2C), more severe than would be fitted by the effects of height mediated by GA-insensitivity. When tillage intensity was reduced, however, (Fig 3H and 3I), *Rht12* produced greater grain specific weights than *Rht-D1c*, contributing to a significant (*P<*0.05) tillage x allele interaction.

### Hagberg falling number

When averaged over the six years of the conventional experiments in Series 1, there was a clear linear increase in Hagberg falling number as canopy height was reduced with GA-insensitivity (Fig 1H). This effect, however, was not evident in all years (Fig 2H). In the tillage experiments the *Rht-D1c* NIL in Mercia had significantly (*P*<0.05) poorer falling numbers than did the slightly taller *Rht-B1c* allele such that the fit with height justified the inclusion of a quadratic effect (Fig 3J–3L). In Series 4 HFN was increased by nitrogen application, *Rht-B1b* and *Rht-B1c* (Fig 4G and 4H).

In contrast to the effect of *Rht-X1x* alleles, dwarfing with GA-sensitive alleles was never associated with improved Hagberg falling number (Figs 1H, 2H and 3J–3L; Table 2).

### Nitrogen concentration

**Fig 5:**
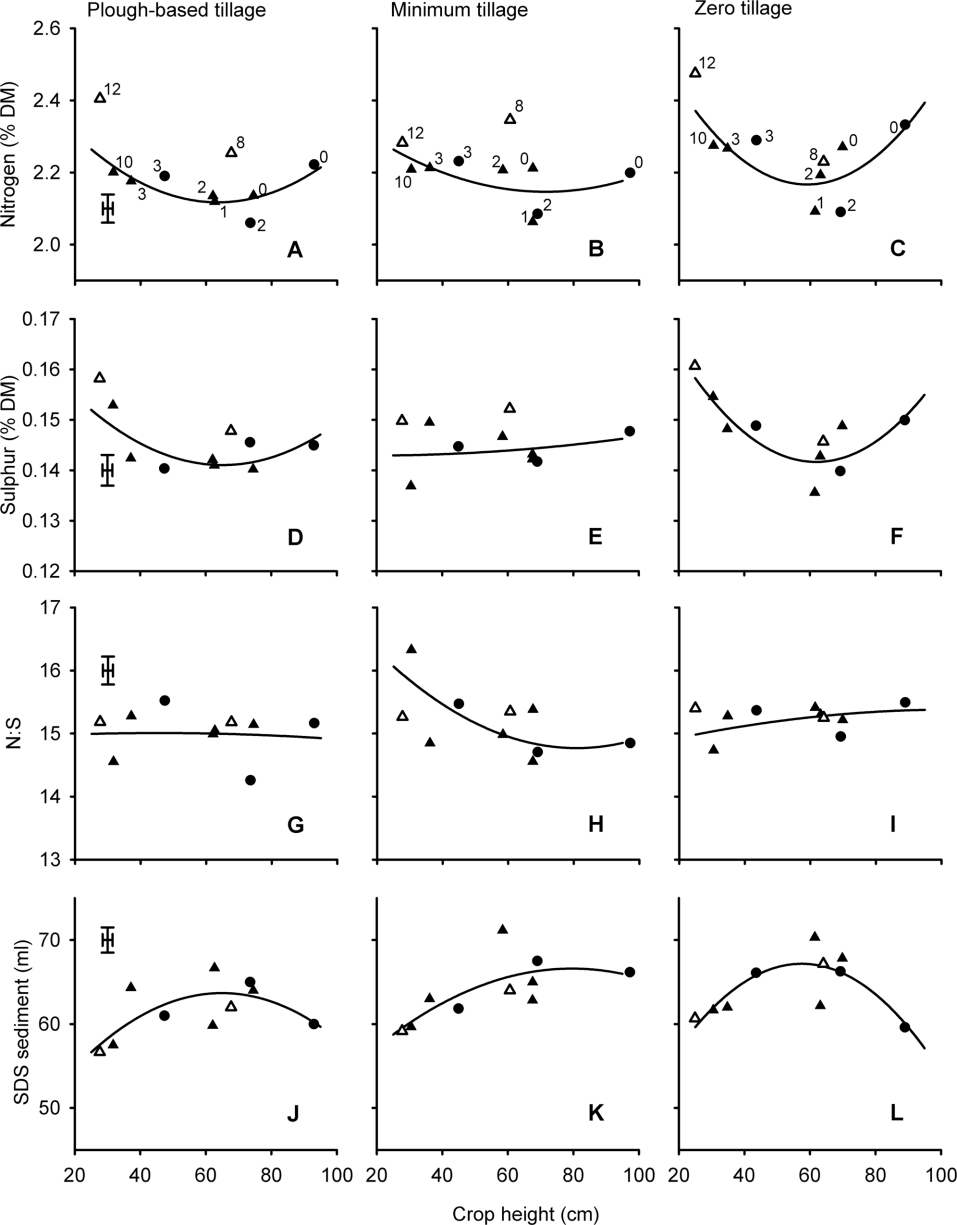
*Rht* and tillage effects on nitrogen, sulphur and SDS-sediment of wheat related to crop height. Numbers in panels A-C correspond to *Rht-* alleles (0 = tall, 1 = *B1b*, 2 = *D1b*, 3 = *B1c*, 8 = *Rht8* + *Ppd-D1a*, 10 = *D1c*, 12 = *Rht12*) in Mercia (▲, △), and Maris Widgeon (●) backgrounds. Fits are quadratic. Open symbols (8 = *Rht8* + *Ppd-D1a*, 12 = *Rht12*) are gibberellin-sensitive dwarfing alleles and not included in the fits. Alleles in all other panels can be inferred from labelling in panels A-C as heights of alleles are consistent. Main effects of background on the fitted constant have been removed from all data points. Points are means from 3 blocks in each of two years. Error bars are one standard error of difference for comparing alleles within tillage treatment (54 D.F.).

The effects of *Rht* allele on nitrogen (or crude protein) concentration were usually the opposite to those of yield. i.e. the effects of *Rht-X1x* allele could generally be described with a quadratic fit, with a minimum occurring around a height of 80 cm in the conventionally-managed series (Fig 1D), or slightly lower in the tillage series (Fig 5A–5C). In the organic context there was no dilution of nitrogen as heights declined to 80 cm (Fig 2D), but here there was no increase in yield either (Fig 2A).

In Series 4, *Rht-B1b* again reduced nitrogen concentration, particularly at high N rates (Fig 4I) at which the allele had also been most effective at increasing grain yield. In 2011, however, both *Rht-B1b* and *Rht-B1c* diluted nitrogen concentration without increasing yield (Fig 4J).

As with the GA-insensitive alleles, effects of *Rht8+Ppd-D1a* and *Rht12* on grain nitrogen concentration tended to be in the opposite direction to effects on yield. For example *Rht8*+*Ppd-D1a* increased nitrogen concentration compared to that achieved by GA-insensitive NILs of similar height in the conventional series (Fig 1D), but not when grown organically (Fig 2D) or, apparently, after zero tillage (Fig 5C). In contrast, in the Paragon lines (Table 2) *Rht8* reduced grain nitrogen concentration at the highest N rate, despite also reducing yield at the same level of fertilization.

### Sulphur concentration and nitrogen: sulphur ratio

Effects of alleles, height and tillage on grain sulphur concentration tended to follow the effects already described for grain nitrogen concentration (Figs 1E, 2E and 5D–5F), except that nitrogen concentration was slightly more responsive to height and nitrogen fertilizer application. The greater relative effects of treatments on N compared with S concentrations therefore resulted in N:S ratios reaching a minimum at heights around 80 cm in the conventional management (Fig 1F), increasing with dwarfism in the organic context (Fig 2F), and increasing with nitrogen application rate (Fig 4M and 4N). In the nitrogen response experiments there was no effect of Allele on N:S ratios in 2010 (Fig 4M). In 2012, however, the dilution in nitrogen concentration associated with the dwarfing alleles (Fig 4J) was not matched in sulphur concentration (Fig 4L) such that N:S ratio was consistently reduced (i.e. improved) by *Rht-B1b* (Fig 4N).

The N:S ratios from the GA-sensitive alleles *Rht8*+*Ppd-D1a* or *Rht12* were not significantly (*P*>0.05) different from GA-insensitive alleles conferring comparable heights (Figs 1F, 2F and 5G–5I). *Rht8* in Paragon had no significant effect on N:S ratio (Table 2)

### SDS-sedimentation volume

The response of SDS-sedimentation volume to crop height as modified by *Rht-X1x* alleles in the conventional series (Fig 1G) and in the tillage experiments (Fig 5J–5L) contrasted with the response of nitrogen concentration (Fig 1D; Fig 5A–5C): i.e. rather than showing a trough at around 80 cm, SDS-sedimentation tended to benefit from semi-dwarfism when it increased grain yield. In the organic experiments there was a clear benefit of dwarfing on SDS-sedimentation volume (Fig 2G).

Nitrogen fertilizer application increased SDS-sedimentation volume in 2010, but not in 2011 (Fig 4O and 4P). In 2010 there was no main effect of Allele on SDS-sedimentation volume; in 2011, dwarfing reduced SDS sedimentation volume although for *Rht-B1c* this was only significant at low N rates (Fig 4P).

With regards to the GA-sensitive alleles, in the conventional series *Rht8* + *Ppd-D1a* failed to increase SDS-sedimentation volume despite increasing both nitrogen and sulphur concentration compared with the effect *Rht-X1x* conferring similar heights (Fig 1G). *Rht12* gave the least volume of SDS-sediment despite having the highest nitrogen and sulphur concentration of any NIL. In the organic and tillage experiments, *Rht8* + *Ppd-D1a* and *Rht12* gave similar SDS sedimentation volumes to *Rht-X1x* NILs of comparable height (Fig 2G). When *Rht8* was isolated from *Ppd-D1a* in the Paragon lines the relevant NIL had significantly reduced SDS-sedimentation volume compared with Paragon, but not compared with the tall backcross (Table 2).

## Discussion

### Grain yield

The utility of *Rht-X1x* alleles for optimizing crop height for grain yield is confirmed [6], as is the quadratic nature of the response over the range of heights observed [6–7]. Excessive dwarfing with impaired GA-sensitivity can be associated with reduced: interception of photosynthetically active radiation, radiation-use efficiency, above ground biomass, grains per ear, and harvest index (HI) [7]; and increased disease [34] and possibly also canopy temperature [35]. Optimal heights are associated with increased grains per ear and improved harvest index, sufficient to counter any reductions in above ground biomass [6–7, 36, 37]. Excessive heights lead to reduced grains per ear and harvest index, and increased lodging at commercially-relevant application rates of nitrogen fertilizer [7].

The benefits of adding semi-dwarfing alleles in otherwise tall lines (>1m) was insignificant in the organic context. Here there was a negative relationship between height and late-season weed prevalence [7] and lodging was minimal in the absence of any synthetic nitrogen application. In individual organic experiments with apparently less intense late-season competition from weeds, semi-dwarfing with *Rht-B1b* or *Rht-D1b* can increase grain yield [38] but the results from Series 4 confirm that the benefits of semi-dwarfing for yield, at least with *Rht-B1b*, are more likely to be expressed at higher levels of nitrogen availability [39]. We also demonstrate that the yield penalty conferred by the severe dwarfs such as *Rht-B1c* cannot be rectified by applying more nitrogen fertilizer during stem extension. In a high nitrogen context we find no strong evidence that optimum height, as influenced by *Rht-X1x* alleles, varies with tillage intensity.

We demonstrate a clear yield penalty for both *Rht8* + *Ppd-D1a*, and *Rht8* alone at commercially-relevant levels of nitrogen fertilizer and after plough-based tillage [7, 30]. In combination with *Ppd-D1a* light interception is curtailed by earlier senescence [7] whilst for *Rht8* alone reduced light interception after anthesis appears to be related to effects on canopy size [30]. That these deficiencies were not reflected in reduced yield in the zero-tillage is consistent with observations that GA-sensitivity is more desirable in challenging establishment conditions [11, 40]. The lack of a yield penalty for *Rht8* + *Ppd-D1a* in the organic context appears to be more a reflection of the benefits of earliness conferred by *Ppd-D1a* for nitrogen capture and possibly weed completion, rather than an effect of Rht8 [7].

### Mean grain weight and grain specific weight

We confirm the negative relationship between the potency of *Rht-X1x* alleles for reducing height and their effect on mean grain weight [6, 38, 41]. We also demonstrate a level of variation around this relationship which would accommodate contrary reports of, for example, *Rht-B1c* occasionally increasing mean grain weight [42]. Flintham et al. [6,9] ascribe reductions in mean grain weight to increased competition for post-anthesis assimilate because in their study *Rht-X1x* dwarfing alleles also increased grains per spikelet. Miralles et al.[43] provide further analysis of the implications of assimilate supply: where for *Rht-B1b and Rht-D1b* more of the distal florets in the spikelet survived to anthesis; grains set in these distal sites, however, had lower potential grain weights compared to the proximal grain sites; grain samples from the taller lines had a greater proportion from proximal sites because distal florets had aborted. An additional complicating factor is that we now know that assimilate supply before anthesis can influence potential mean grain weight through effects on carpel size [44].

In contrast to the situation with semi-dwarfing [6,9,43], in these experiments severe dwarfing with either *Rht-B1c* or *Rht-D1c* in Mercia reduced both mean grain weight and grain numbers per ear simultaneously. Concurrent reductions in grain numbers and mean grain weights were also reported by Appleford et al. [10] who produced dwarfed plants associated with decreased content of bioactive gibberellins in transgenic wheat. Simultaneous reductions in mean grain weight and grain numbers do not necessarily rule out arguments centred on assimilate supply but it is possible that GA signalling directly interferes with grain size development. Appleford et al. [10] suggest a possible direct involvement of GA citing evidence from other plant species. However, recent studies [45] on wheat have failed to identify GA_1+4_ activity as being correlated with grain filling rate or duration. That direct influences of GA signalling is the main mechanism by which the *Rht-X1x* alleles influence mean grain weight is not supported by this study because of the degree of scatter around the relationship with height and the occasional large grains resulting from severe dwarfs such as the report for *Rht-B1c* mentioned previously [42], and for example Maris Huntsman + *Rht-D1b* + *Rht-B1b* reported here in Series 1. Some direct involvement of GA in grain size may, however, explain the particularly small grains observed for *Rht12* if, as suggested [17], *Rht12* interferes with GA biosynthesis. We confirm the detrimental effect of *Rht12* on mean grain weight [17,46], but additionally demonstrate that this is often in excess of that predicted by the effect of crop height estimated from the relationship with *Rht-X1x* alleles. Despite this ‘extra’ detrimental effect of *Rht12*, others [46] have still explained the small grains from *Rht12* as being a result of inadequate post-anthesis assimilate supply from the very short plants, rather than propose direct effects of GA deficiency.

We show that specific weight, the packing density of grain, can be negatively and closely related to reductions in crop height as manipulated by *Rht-X1x* alleles. Negative effects have previously been reported for *Rht-B1b* [47–49], *Rht-*D1b [41,48,50], and *Rht-B1b*+*Rht-D1b* [38] but we demonstrate these to be part of an apparently continuous response to height and degree of GA-insensitivity that is conserved over contrasting production systems and tillage intensities. It is notable that the relationship between specific weight and height is usually closer than that between mean grain weight and height. It is possible that specific weight is being influenced more by grain shape and shrivelling factors and less influenced by grain size *per se* and confounding influences of potential grain weight. Packing density is, however, a complex trait influenced by individual grain density and grain surface characteristics as well as grain shape and shrivelling [19]. Our results do not support a direct pleiotropic effect of *Rht-X1x* alleles on grain specific weight as *Rht8*+*Ppd-D1a* gave similar values to that predicted for the relationship with height, and *Rht8* alone also reduced grain specific weight. As with mean grain weight, *Rht12* often gave markedly lower specific weights than would be predicted from the height relationships based on *Rht-X1x* alleles, and at least in this case visual inspection of the grain would suggest that grain shrivelling was a major contributor to poor packing density.

It is clear that reductions in mean grain weight and grain specific weight by *Rht-B1x* alleles, or specific weight by *Rht8*, cannot be countered by increasing nitrogen fertilizer applications during stem extension.

### Hagberg falling number

We confirm the benefits of the *Rht-X1x* dwarfing alleles for increasing the Hagberg falling number of wheat [9,43], associated with reductions in *alpha*-amylase activity. Series 1 demonstrates a broadly linear, positive relationship between effects of *Rht-X1x* alleles on height, and their effects on Hagberg falling number. However, results from Series 1, 3 and 5 strongly suggests that the effect of *Rht-X1x* is a pleiotropic influence not mediated through direct effects on height because the GA-sensitive lines *Rht8*, Rht8+*PpdD1a* and *Rht12* failed to increase falling number. GA-sensitivity and signalling is heavily implicated in the production of *alpha*-amylase both before [51] and after [52] the onset of germination and both sources of the enzyme can be reduced by *Rht-X1x* alleles [53, 54]. The observation that *Rht-B1c* was more potent than *Rht-D1c* for increasing falling numbers in Series 3, despite *Rht-D1c* producing the shorter plants may well be due to *Rht-B1c* having the greater effect on grain dormancy [53]. The benefit for *Rht-X1x* alleles is independent of tillage intensity and nitrogen application.

### Nitrogen and sulphur concentration

Results here are consistent with previous results showing that when semi-dwarfing *Rht-X1x* alleles increase yield, they also reduce grain nitrogen concentration [37–38, 50]. This is in spite of total nitrogen yields increasing when grain yields are increased [39]. In some previous studies nitrogen concentration appears to have been reduced even when grain yields were apparently unaffected, leading to the suggestion that nitrogen dilution is a direct pleiotropic effect of the *Rht-X1x* alleles [8]. We also found in, two of the fourteen field experiments, situations where both GA-insenstive (in Series 4) and GA sensitive (in Series 5) Rht alleles diluted grain nitrogen concentration without increasing yield. Dwarfing can impede nitrogen uptake into the above ground biomass after anthesis [36], and it is this late nitrogen that can have a disproportionate effect on grain protein concentration [55]. We do not, however, have strong evidence that protein dilution by *Rht-X1x* alleles can be attributed to a direct pleiotropic effect: dilution was achieved with both GA-sensitive and GA-insensitive alleles; in the organic experiments none of the *Rht* alleles reduced nitrogen concentration; and in the conventional experiments severe dwarfing often increased grain nitrogen concentration relative to alleles giving optimal heights for yield. Further, the relationships between grain nitrogen concentration and grain yield in Series 1 and Series 2 are less negative than those reported for similarly yielding crops from a review of 106 variety evaluations in field experiments [56].

We demonstrate here that when semi-dwarfing alleles reduce grain nitrogen concentration, they do not necessarily reduce grain sulphur concentration to the same extent such that grain N:S ratios can sometimes be improved (i.e. reduced) with semi-dwarfing alleles. SDS-sedimentation volume is also often improved by *Rht* alleles. In some cases, such as in the organic system, improvements in SDS are consistent with effects on nitrogen and sulphur concentrations. In Series 1 and 3, however, effects of *Rht* alleles on SDS-sedimentation volume appear to counter effects on N and S concentrations, and in Series 1, better related to (the inverse of) N:S ratio. It has been previously been shown that SDS-sedimentation volume can be reduced by *Rht-B1b* and *Rht-D1b* commensurate with a reduction in nitrogen concentration [44]. Others [57] have found no effect of *Rht-B1c* on SDS-sedimentation volume despite a reduction in nitrogen concentration as was the case here. i.e. usually when *Rht-X1x* had diluted nitrogen or sulphur concentrations relative to *rht* (tall) there was no commensurate reduction in SDS-sedimentation volume.

## Conclusions

Semi-, or severe- dwarfing with *Rht-X1x* alleles reduces mean grain weight and grain specific weight and increases Hagberg falling number irrespective of system (‘conventional’ *vs* ‘organic’), tillage intensity or nitrogen application rate. Only for the effect on Hagberg falling number is their strong evidence for a direct pleiotropic effect of GA-insenstivity. Effects of *Rht-X1x* on grain specific weight and mean grain weight can be replicated by GA-sensitive alleles and may be explained on the basis of probable changes in assimilate supply before and after anthesis influencing grain numbers, potential grain weight, and the ability to fill grains. Effects of *Rht* (GA-sensitive and GA-insensitive) alleles on grain nitrogen and sulphur concentrations can mostly be explained by close and negative relationships with effects on grain yield. However, grain nitrogen can sometimes be diluted by dwarfing alleles in the absence of yield increases. When nitrogen dilution does occur sulphur is not necessarily diluted to the same extent such that N:S ratio decreases. The inference from the HFN, N:S and SDS-sedimentation volume results are that, even when *Rht*-*X1x* have reduced grain nitrogen concentrations any negative effect on loaf quality could be mitigated by stability or improvements in other relevant criteria.

## Supporting Information

S1 Table Effect of cultivar background and dwarfing allele in near-isogenic lines of winter wheat grown conventionally on crop height, and grain yield and quality. (DOCX)

S2 Table Effect of cultivar background and dwarfing allele in near-isogenic lines of winter wheat grown organically on crop height, and grain yield and quality. (DOCX)

S3 Table Effect of cultivar background and dwarfing allele in near-isogenic lines of winter wheat established with different tillage intensities on crop height, and grain yield and quality. (DOCX)

S4 Table Effect of cultivar background and dwarfing allele in near-isogenic lines of winter wheat grown with different levels of nitrogen fertilizer applications on grain yield and quality in 2010. (DOCX)

S5 Table Effect of cultivar background and dwarfing allele in near-isogenic lines of winter wheat grown with different levels of nitrogen fertilizer applications on grain yield and quality in 2011. (DOCX)
