# Supplementary material for: Reduced Height (Rht) Alleles Affect Wheat Grain Quality
Source: PLoS One. 2016 May 19;11(5):e0156056. doi: 10.1371/journal.pone.0156056 (PMC4873232; doi:10.1371/journal.pone.0156056)
Supplement: S1 Table — (DOCX) [file pone.0156056.s001.docx]

| Background and dwarfing allele | Crop height (cm) | Grain yield  (t DM/ha) | Mean grain weight (mg DM) | Grain specific weight (kg/hl) | Hagberg falling number | Grain N conc. (% DM) | Grain S conc. (% DM) | Grain N:S ratio | SDS sediment volume (ml) |
| --- | --- | --- | --- | --- | --- | --- | --- | --- | --- |
| Mercia |  |  |  |  |  |  |  |  |  |
| *rht*(tall) | 81.0 | 6.60 | 41.6 | 78.4 | 329 | 2.40 | 0.156 | 15.4 | 65.5 |
| *Rht-B1b* | 73.5 | 6.61 | 38.5 | 76.8 | 323 | 2.34 | 0.156 | 15.1 | 61.1 |
| *Rht-D1b* | 69.1 | 6.12 | 36.4 | 75.6 | 342 | 2.33 | 0.155 | 15.0 | 68.0 |
| *Rht-B1c* | 44.0 | 4.99 | 32.2 | 71.5 | 376 | 2.42 | 0.157 | 15.4 | 62.3 |
| *Rht-D1c* | 36.0 | 3.33 | 32.0 | 69.4 | 364 | 2.58 | 0.171 | 15.2 | 61.0 |
| *Rht8* | 72.1 | 5.21 | 38.5 | 76.2 | 269 | 2.48 | 0.163 | 15.2 | 60.3 |
| *Rht12* | 32.2 | 2.45 | 27.2 | 65.1 | 268 | 2.82 | 0.179 | 15.7 | 56.4 |
| Maris Huntsman |  |  |  |  |  |  |  |  |  |
| *rht*(tall) | 97.7 | 5.81 | 48.9 | 75.2 | 205 | 2.54 | 0.161 | 15.8 | 51.3 |
| *Rht-B1b* | 79.6 | 6.76 | 45.5 | 74.6 | 255 | 2.32 | 0.154 | 15.0 | 52.5 |
| *Rht-D1b* | 77.3 | 6.68 | 45.8 | 73.5 | 264 | 2.26 | 0.150 | 15.2 | 50.9 |
| *Rht-B1c* | 45.6 | 4.90 | 41.8 | 69.5 | 325 | 2.49 | 0.160 | 15.6 | 53.5 |
| *Rht-B1b+D1b* | 55.2 | 5.53 | 43.2 | 71.3 | 260 | 2.46 | 0.159 | 15.5 | 57.1 |
| *Rht-B1c+D1b* | 37.9 | 3.41 | 46.1 | 69.5 | 323 | 2.77 | 0.174 | 16.0 | 51.6 |
| Maris Widgeon |  |  |  |  |  |  |  |  |  |
| *rht*(tall) | 100.8 | 4.30 | 46.8 | 77.6 | 218 | 2.76 | 0.177 | 15.6 | 74.9 |
| *Rht-B1b* | 82.6 | 5.61 | 42.2 | 76.6 | 278 | 2.52 | 0.162 | 15.7 | 76.5 |
| *Rht-D1b* | 82.6 | 5.80 | 43.9 | 76.1 | 282 | 2.52 | 0.167 | 15.2 | 79.6 |
| *Rht-B1c* | 53.4 | 4.52 | 41.5 | 72.4 | 344 | 2.73 | 0.175 | 15.7 | 75.3 |
| *Rht-B1b+D1b* | 55.5 | 4.46 | 37.1 | 72.0 | 275 | 2.60 | 0.168 | 15.6 | 79.0 |
| *Rht-B1c+D1b* | 47.2 | 3.36 | 41.6 | 72.5 | 310 | 2.69 | 0.171 | 15.8 | 75.1 |
| Average SED (>300 d.f.) | |  |  |  |  |  |  |  |  |
|  | 1.49 | 0.287 | 1.09 | 0.64 | 14.8 | 0.044 | 0.0033 | 0.23 | 2.09 |
| SED for comparing Mercia alleles | | |  |  |  |  |  |  |  |
|  | 1.18 | 0.233 | 0.88 | 0.52 | 11.9 | 0.034 | 0.0025 | 0.18 | 1.63 |
| Maximum SED |  |  |  |  |  |  |  |  |  |
|  | 1.71 | 0.324 | 1.23 | 0.77 | 17.8 | 0.053 | 0.0040 | 0.28 | 2.56 |
