# Supplementary material for: Reduced Height (Rht) Alleles Affect Wheat Grain Quality
Source: PLoS One. 2016 May 19;11(5):e0156056. doi: 10.1371/journal.pone.0156056 (PMC4873232; doi:10.1371/journal.pone.0156056)
Supplement: S2 Table — (DOCX) [file pone.0156056.s002.docx]

| Background and dwarfing allele | Crop height (cm) | Grain yield  (t DM/ha) | Mean grain weight (mg DM) | Grain specific weight (kg/hl) | Hagberg falling number | Grain N conc.  (% DM) | Grain S conc.  (% DM) | Grain N:S ratio | SDS sediment volume (ml) |
| --- | --- | --- | --- | --- | --- | --- | --- | --- | --- |
| Mercia |  |  |  |  |  |  |  |  |  |
| *rht*(tall) | 89.2 | 3.63 | 34.9 | 75.4 | 292 | 1.76 | 0.123 | 14.3 | 47.5 |
| *Rht-B1b* | 82.1 | 3.52 | 31.3 | 73.3 | 274 | 1.80 | 0.128 | 14.1 | 46.1 |
| *Rht-D1b* | 76.9 | 2.65 | 29.0 | 70.6 | 277 | 1.82 | 0.135 | 13.5 | 50.7 |
| *Rht-B1c* | 45.4 | 1.32 | 22.2 | 62.0 | 313 | 2.21 | 0.151 | 14.5 | 50.2 |
| *Rht-D1c* | 36.2 | 0.77 | 26.2 | 63.1 | 258 | 2.36 | 0.158 | 14.9 | 50.7 |
| *Rht8* | 78.4 | 3.06 | 32.0 | 72.8 | 240 | 1.78 | 0.134 | 13.5 | 42.9 |
| *Rht12* | 30.3 | 0.30 | 18.9 | 52.7 | 215 | 2.66 | 0.173 | 15.3 | 48.7 |
| Maris Huntsman |  |  |  |  |  |  |  |  |  |
| *rht*(tall) | 115.5 | 3.16 | 44.3 | 71.5 | 202 | 1.73 | 0.121 | 14.2 | 32.0 |
| *Rht-B1b* | 88.5 | 3.55 | 40.7 | 71.3 | 218 | 1.75 | 0.123 | 14.2 | 29.6 |
| *Rht-D1b* | 87.8 | 4.05 | 38.4 | 69.7 | 264 | 1.75 | 0.121 | 14.3 | 35.6 |
| *Rht-B1c* | 51.8 | 2.40 | 31.3 | 64.5 | 226 | 2.01 | 0.136 | 14.9 | 37.0 |
| *Rht-B1b+D1b* | 61.8 | 1.87 | 35.8 | 66.6 | 198 | 2.07 | 0.138 | 15.1 | 38.3 |
| *Rht-B1c+D1b* | 43.8 | 1.33 | 37.1 | 65.1 | 222 | 2.28 | 0.156 | 15.0 | 57.0 |
| Maris Widgeon |  |  |  |  |  |  |  |  |  |
| *rht*(tall) | 110.2 | 2.48 | 44.4 | 75.2 | 239 | 2.04 | 0.150 | 13.9 | 61.3 |
| *Rht-B1b* | 89.5 | 2.67 | 41.0 | 75.6 | 276 | 2.02 | 0.144 | 14.2 | 75.0 |
| *Rht-D1b* | 99.8 | 2.90 | 44.9 | 74.0 | 224 | 2.13 | 0.142 | 15.2 | 73.0 |
| *Rht-B1c* | 62.5 | 2.33 | 37.7 | 70.3 | 243 | 2.56 | 0.167 | 15.8 | 70.0 |
| *Rht-B1b+D1b* | 64.8 | 2.03 | 40.0 | 72.3 | 206 | 2.23 | 0.159 | 14.5 | 80.0 |
| *Rht-B1c+D1b* | 51.2 | 1.52 | 40.7 | 70.5 | 200 | 2.54 | 0.168 | 15.6 | 75.0 |
| Average SED (84 d.f.) | |  |  |  |  |  |  |  |  |
|  | 2.92 | 0.478 | 1.59 | 2.04 | 23.8 | 0.132 | 0.0092 | 0.69 | 5.17 |
| SED for comparing Mercia alleles | | |  |  |  |  |  |  |  |
|  | 1.78 | 0.291 | 0.97 | 1.22 | 14.5 | 0.080 | 0.0057 | 0.42 | 3.15 |
| Maximum SED |  |  |  |  |  |  |  |  |  |
|  | 3.41 | 0.558 | 1.85 | 2.62 | 27.7 | 0.153 | 0.0109 | 0.81 | 6.03 |
