# Supplementary material for: Reduced Height (Rht) Alleles Affect Wheat Grain Quality
Source: PLoS One. 2016 May 19;11(5):e0156056. doi: 10.1371/journal.pone.0156056 (PMC4873232; doi:10.1371/journal.pone.0156056)
Supplement: S3 Table — (DOCX) [file pone.0156056.s003.docx]

| Tillage, background and dwarfing allele | Crop height (cm) | Grain yield  (t DM/ha) | Mean grain weight (mg DM) | Grain specific weight (kg/hl) | Hagberg falling number | Grain N conc.  (% DM) | Grain S conc.  (% DM) | Grain N:S ratio | SDS sediment volume (ml) |
| --- | --- | --- | --- | --- | --- | --- | --- | --- | --- |
| Plough-based tillage | |  |  |  |  |  |  |  |  |
| Mercia |  |  |  |  |  |  |  |  |  |
| rht(tall) | 74.5 | 7.76 | 46.5 | 75.2 | 314 | 2.14 | 0.140 | 15.1 | 64.0 |
| Rht-B1b | 62.1 | 7.45 | 42.3 | 73.3 | 322 | 2.14 | 0.142 | 15.0 | 59.8 |
| Rht-D1b | 62.6 | 7.73 | 41.5 | 72.8 | 327 | 2.12 | 0.141 | 15.0 | 66.7 |
| Rht-B1c | 37.1 | 5.71 | 35.5 | 69.0 | 377 | 2.18 | 0.142 | 15.3 | 64.3 |
| Rht-D1c | 31.7 | 5.10 | 32.9 | 65.7 | 341 | 2.20 | 0.153 | 14.6 | 57.5 |
| Rht8 | 67.7 | 5.77 | 43.7 | 73.1 | 271 | 2.25 | 0.148 | 15.2 | 62.0 |
| Rht12 | 27.6 | 3.64 | 32.1 | 65.9 | 241 | 2.41 | 0.158 | 15.2 | 56.7 |
| Maris Widgeon |  |  |  |  |  |  |  |  |  |
| rht(tall) | 93.0 | 5.63 | 52.5 | 74.7 | 232 | 2.46 | 0.156 | 15.7 | 77.8 |
| Rht-D1b | 73.5 | 6.68 | 48.3 | 73.7 | 269 | 2.29 | 0.156 | 14.8 | 82.8 |
| Rht-B1c | 47.4 | 6.06 | 43.7 | 68.8 | 328 | 2.42 | 0.151 | 16.1 | 78.8 |
| Minimum tillage | |  |  |  |  |  |  |  |  |
| Mercia |  |  |  |  |  |  |  |  |  |
| rht(tall) | 67.6 | 5.47 | 45.8 | 74.0 | 332 | 2.21 | 0.143 | 15.4 | 65.0 |
| Rht-B1b | 67.6 | 6.68 | 41.2 | 72.7 | 294 | 2.06 | 0.142 | 14.6 | 62.8 |
| Rht-D1b | 58.4 | 5.17 | 40.5 | 71.8 | 355 | 2.21 | 0.147 | 15.0 | 71.2 |
| Rht-B1c | 36.1 | 4.95 | 34.5 | 68.1 | 383 | 2.21 | 0.150 | 14.8 | 63.0 |
| Rht-D1c | 30.4 | 4.64 | 34.9 | 65.7 | 303 | 2.21 | 0.137 | 16.3 | 59.7 |
| Rht8 | 60.6 | 4.18 | 41.7 | 72.2 | 270 | 2.35 | 0.152 | 15.3 | 64.0 |
| Rht12 | 27.7 | 3.92 | 34.9 | 68.8 | 220 | 2.28 | 0.150 | 15.3 | 59.2 |
| Maris Widgeon |  |  |  |  |  |  |  |  |  |
| rht(tall) | 97.3 | 4.38 | 50.9 | 74.2 | 218 | 2.50 | 0.160 | 15.6 | 80.2 |
| Rht-D1b | 69.0 | 4.42 | 44.8 | 71.6 | 289 | 2.39 | 0.154 | 15.4 | 81.5 |
| Rht-B1c | 45.0 | 4.32 | 42.9 | 69.5 | 335 | 2.53 | 0.157 | 16.2 | 75.8 |
| Zero tillage | |  |  |  |  |  |  |  |  |
| Mercia |  |  |  |  |  |  |  |  |  |
| rht(tall) | 69.9 | 5.30 | 47.2 | 76.4 | 321 | 2.27 | 0.149 | 15.2 | 67.8 |
| Rht-B1b | 63.2 | 5.09 | 41.9 | 73.2 | 341 | 2.19 | 0.143 | 15.3 | 62.2 |
| Rht-D1b | 61.5 | 5.97 | 40.0 | 71.5 | 362 | 2.09 | 0.136 | 15.4 | 70.3 |
| Rht-B1c | 34.8 | 4.59 | 35.5 | 68.3 | 375 | 2.27 | 0.148 | 15.3 | 62.0 |
| Rht-D1c | 30.5 | 3.84 | 33.5 | 64.5 | 323 | 2.28 | 0.155 | 14.7 | 61.7 |
| Rht8 | 64.1 | 5.42 | 41.4 | 71.9 | 250 | 2.23 | 0.146 | 15.2 | 67.2 |
| Rht12 | 25.0 | 3.45 | 34.7 | 69.5 | 263 | 2.48 | 0.161 | 15.4 | 60.7 |
| Maris Widgeon |  |  |  |  |  |  |  |  |  |
| rht(tall) | 89.0 | 4.10 | 49.5 | 74.5 | 239 | 2.58 | 0.162 | 15.9 | 74.2 |
| Rht-D1b | 69.3 | 4.87 | 46.9 | 71.7 | 245 | 2.34 | 0.152 | 15.3 | 80.8 |
| Rht-B1c | 43.6 | 4.20 | 42.6 | 68.8 | 321 | 2.54 | 0.161 | 15.7 | 80.7 |
| SED for comparisons within Tillage levels (54 d.f.) | | | | |  |  |  |  |  |
|  | 3.05 | 0.703 | 1.35 | 0.99 | 24.1 | 0.079 | 0.0061 | 0.44 | 3.00 |
| SED for comparisons across Tillage levels | | | |  |  |  |  |  |  |
|  | 2.93 | 0.770 | 1.45 | 0.98 | 24.5 | 0.082 | 0.0065 | 0.44 | 2.98 |
