# Supplementary material for: Reduced Height (Rht) Alleles Affect Wheat Grain Quality
Source: PLoS One. 2016 May 19;11(5):e0156056. doi: 10.1371/journal.pone.0156056 (PMC4873232; doi:10.1371/journal.pone.0156056)
Supplement: S4 Table — (DOCX) [file pone.0156056.s004.docx]

| Background and dwarfing allele | N rate (kg N/ha) | Grain yield  (t DM/ha) | Mean grain weight (mg DM) | Grain specific weight (kg/hl) | Hagberg falling number | Grain N conc.  (% DM) | Grain S conc.  (% DM) | Grain N:S ratio | SDS sediment volume (ml) |
| --- | --- | --- | --- | --- | --- | --- | --- | --- | --- |
| Mercia |  |  |  |  |  |  |  |  |  |
| rht(tall) | 0 | 3.76 | 43.2 | 75.2 | 261 | 1.24 | 0.101 | 12.3 | 37.0 |
|  | 100 | 6.93 | 40.9 | 77.3 | 320 | 1.54 | 0.107 | 14.4 | 58.0 |
|  | 200 | 6.95 | 40.2 | 76.9 | 354 | 1.97 | 0.125 | 15.7 | 68.7 |
|  | 350 | 7.27 | 38.4 | 75.5 | 395 | 2.42 | 0.140 | 17.3 | 69.3 |
| Rht-B1b | 0 | 4.38 | 43.2 | 76.2 | 302 | 1.39 | 0.107 | 13.0 | 42.3 |
|  | 100 | 7.33 | 42.8 | 74.4 | 303 | 1.52 | 0.106 | 14.3 | 52.7 |
|  | 200 | 8.90 | 42.1 | 75.5 | 325 | 1.80 | 0.116 | 15.4 | 63.7 |
|  | 350 | 10.00 | 41.5 | 75.6 | 361 | 2.20 | 0.129 | 17.0 | 71.7 |
| Rht-B1c | 0 | 4.12 | 38.3 | 73.3 | 339 | 1.30 | 0.105 | 12.4 | 40.7 |
|  | 100 | 6.18 | 36.8 | 71.9 | 344 | 1.48 | 0.103 | 14.4 | 49.0 |
|  | 200 | 7.10 | 33.2 | 72.0 | 391 | 1.94 | 0.122 | 15.9 | 64.7 |
|  | 350 | 7.00 | 32.4 | 70.3 | 407 | 2.48 | 0.145 | 17.1 | 72.3 |
| Maris Huntsman |  |  |  |  |  |  |  |  |  |
| rht(tall) | 0 | 4.04 | 49.4 | 73.1 | 99 | 1.31 | 0.096 | 13.6 | 23.7 |
|  | 100 | 6.87 | 49.5 | 75.1 | 172 | 1.62 | 0.103 | 15.8 | 41.0 |
|  | 200 | 7.97 | 49.3 | 75.4 | 200 | 1.95 | 0.121 | 16.1 | 54.7 |
|  | 350 | 8.53 | 49.8 | 75.6 | 230 | 2.30 | 0.135 | 17.0 | 56.3 |
| Rht-B1b | 0 | 4.63 | 47.2 | 74.3 | 177 | 1.23 | 0.103 | 12.0 | 26.0 |
|  | 100 | 6.87 | 45.1 | 74.5 | 239 | 1.46 | 0.097 | 15.0 | 38.3 |
|  | 200 | 8.57 | 45.3 | 72.6 | 310 | 1.92 | 0.122 | 15.8 | 54.7 |
|  | 350 | 9.48 | 44.8 | 74.9 | 290 | 2.18 | 0.125 | 17.4 | 60.0 |
| Rht-B1c | 0 | 4.04 | 43.2 | 71.9 | 331 | 1.29 | 0.091 | 14.2 | 32.7 |
|  | 100 | 6.80 | 41.6 | 70.0 | 346 | 1.55 | 0.101 | 15.3 | 47.7 |
|  | 200 | 7.68 | 42.0 | 70.8 | 379 | 1.98 | 0.123 | 16.1 | 60.3 |
|  | 350 | 7.69 | 41.9 | 71.3 | 444 | 2.48 | 0.140 | 17.7 | 65.7 |
| Maris Widgeon |  |  |  |  |  |  |  |  |  |
| rht(tall) | 0 | 3.57 | 48.9 | 76.9 | 206 | 1.51 | 0.115 | 13.1 | 66.0 |
|  | 100 | 5.81 | 48.7 | 79.6 | 235 | 1.87 | 0.124 | 15.2 | 80.0 |
|  | 200 | 6.97 | 48.7 | 79.7 | 230 | 2.34 | 0.144 | 16.2 | 83.0 |
|  | 350 | 6.92 | 49.4 | 80.3 | 235 | 2.68 | 0.157 | 17.1 | 81.7 |
| Rht-B1b | 0 | 4.53 | 45.5 | 76.0 | 233 | 1.44 | 0.107 | 13.4 | 62.7 |
|  | 100 | 6.94 | 45.1 | 75.0 | 259 | 1.70 | 0.112 | 15.2 | 72.3 |
|  | 200 | 8.18 | 44.0 | 76.4 | 281 | 2.08 | 0.128 | 16.2 | 85.7 |
|  | 350 | 8.22 | 42.6 | 74.5 | 304 | 2.43 | 0.141 | 17.3 | 83.0 |
| Rht-B1c | 0 | 3.86 | 45.4 | 72.8 | 287 | 1.45 | 0.106 | 13.6 | 59.7 |
|  | 100 | 5.52 | 43.8 | 70.0 | 374 | 1.69 | 0.113 | 14.9 | 73.0 |
|  | 200 | 6.25 | 43.7 | 71.0 | 400 | 2.23 | 0.139 | 16.0 | 81.0 |
|  | 350 | 6.36 | 44.2 | 67.7 | 402 | 2.77 | 0.156 | 17.8 | 77.0 |
| SED for comparisons within genotype (54 d.f.) | | | | |  |  |  |  |  |
|  |  | 0.457 | 1.37 | 1.20 | 18.8 | 0.093 | 0.0046 | 0.61 | 3.00 |
| SED for comparisons across genotypes | | | | |  |  |  |  |  |
|  |  | 0.665 | 1.75 | 1.47 | 22.2 | 0.101 | 0.0052 | 0.59 | 4.07 |
