# Supplementary material for: Reduced Height (Rht) Alleles Affect Wheat Grain Quality
Source: PLoS One. 2016 May 19;11(5):e0156056. doi: 10.1371/journal.pone.0156056 (PMC4873232; doi:10.1371/journal.pone.0156056)
Supplement: S5 Table — (DOCX) [file pone.0156056.s005.docx]

| Background and dwarfing allele | N rate (kg N/ha) | Grain yield  (t DM/ha) | Mean grain weight (mg DM) | Grain specific weight (kg/hl) | Hagberg falling number | Grain N conc.  (% DM) | Grain S conc.  (% DM) | Grain N:S ratio | SDS sediment volume (ml) |
| --- | --- | --- | --- | --- | --- | --- | --- | --- | --- |
| Mercia |  |  |  |  |  |  |  |  |  |
| rht(tall) | 0 | 4.34 | 43.0 | 78.9 | 338 | 1.62 | 0.110 | 14.7 | 52.0 |
|  | 50 | 5.27 | 43.2 | 81.1 | 317 | 1.85 | 0.118 | 15.6 | 57.5 |
|  | 100 | 4.81 | 44.5 | 81.1 | 409 | 2.20 | 0.133 | 16.6 | 55.3 |
|  | 200 | 5.65 | 47.0 | 80.5 | 427 | 2.43 | 0.126 | 19.2 | 49.7 |
|  | 350 | 5.88 | 46.7 | 80.4 | 399 | 2.52 | 0.126 | 20.1 | 49.4 |
|  | 450 | 6.01 | 48.0 | 79.9 | 437 | 2.51 | 0.125 | 20.2 | 48.7 |
| Rht-B1b | 0 | 4.10 | 41.9 | 78.1 | 386 | 1.63 | 0.116 | 14.0 | 46.5 |
|  | 50 | 4.19 | 42.7 | 80.6 | 426 | 1.76 | 0.124 | 14.2 | 49.7 |
|  | 100 | 4.44 | 44.5 | 78.7 | 398 | 2.05 | 0.137 | 15.0 | 50.7 |
|  | 200 | 5.08 | 43.6 | 77.8 | 436 | 2.42 | 0.141 | 17.2 | 50.0 |
|  | 350 | 5.82 | 46.7 | 78.7 | 343 | 2.51 | 0.131 | 19.2 | 48.0 |
|  | 450 | 5.68 | 44.4 | 78.4 | 361 | 2.53 | 0.138 | 18.4 | 46.0 |
| Rht-B1c | 0 | 4.66 | 38.6 | 71.5 | 426 | 1.69 | 0.105 | 16.1 | 41.7 |
|  | 50 | 5.21 | 38.1 | 75.4 | 402 | 1.84 | 0.117 | 15.7 | 47.7 |
|  | 100 | 5.54 | 38.7 | 74.0 | 466 | 2.00 | 0.115 | 17.5 | 49.0 |
|  | 200 | 5.87 | 38.8 | 75.7 | 464 | 2.28 | 0.129 | 17.7 | 49.7 |
|  | 350 | 5.74 | 36.1 | 72.7 | 464 | 2.45 | 0.134 | 18.3 | 50.7 |
|  | 450 | 5.47 | 35.1 | 73.1 | 495 | 2.46 | 0.136 | 18.1 | 50.0 |
| Maris Widgeon |  |  |  |  |  |  |  |  |  |
| rht(tall) | 0 | 3.46 | 47.7 | 78.4 | 234 | 1.79 | 0.122 | 14.6 | 67.7 |
|  | 50 | 4.38 | 50.5 | 81.1 | 256 | 2.17 | 0.130 | 16.7 | 68.7 |
|  | 100 | 4.51 | 48.5 | 82.0 | 274 | 2.51 | 0.147 | 17.1 | 69.7 |
|  | 200 | 4.32 | 50.3 | 79.7 | 289 | 2.80 | 0.146 | 19.2 | 67.0 |
|  | 350 | 4.44 | 50.1 | 80.9 | 290 | 2.81 | 0.138 | 20.4 | 65.0 |
|  | 450 | 4.15 | 48.2 | 78.4 | 263 | 2.84 | 0.146 | 19.3 | 63.7 |
| Rht-B1b | 0 | 3.43 | 45.7 | 79.6 | 279 | 1.86 | 0.126 | 14.7 | 64.0 |
|  | 50 | 4.84 | 46.6 | 79.7 | 305 | 2.01 | 0.128 | 15.6 | 70.7 |
|  | 100 | 4.53 | 45.2 | 81.3 | 330 | 2.33 | 0.139 | 16.8 | 69.7 |
|  | 200 | 4.84 | 47.4 | 80.3 | 374 | 2.62 | 0.144 | 18.2 | 65.7 |
|  | 350 | 5.12 | 48.0 | 79.1 | 392 | 2.67 | 0.139 | 19.3 | 59.3 |
|  | 450 | 5.13 | 46.0 | 80.0 | 380 | 2.65 | 0.138 | 19.3 | 58.0 |
| Rht-B1c | 0 | 3.39 | 45.2 | 76.0 | 382 | 1.75 | 0.117 | 15.0 | 58.7 |
|  | 50 | 4.33 | 45.9 | 75.3 | 414 | 1.96 | 0.120 | 16.3 | 68.0 |
|  | 100 | 4.13 | 45.9 | 74.9 | 430 | 2.27 | 0.133 | 17.0 | 68.7 |
|  | 200 | 3.96 | 42.8 | 75.6 | 429 | 2.62 | 0.140 | 18.6 | 67.7 |
|  | 350 | 4.98 | 45.7 | 74.3 | 451 | 2.71 | 0.134 | 20.2 | 62.3 |
|  | 450 | 4.28 | 44.0 | 76.3 | 443 | 2.67 | 0.133 | 20.1 | 58.7 |
| SED for comparisons within genotype (57 d.f.) | | | | | |  |  |  |  |
|  |  | 0.357 | 1.45 | 1.10 | 27.5 | 0.067 | 0.0044 | 0.56 | 2.70 |
| SED for comparisons across genotypes | | | | |  |  |  |  |  |
|  |  | 0.659 | 1.48 | 1.11 | 35.4 | 0.080 | 0.0056 | 0.71 | 2.93 |
